# Preoperative exercise to improve fitness in patients undergoing complex surgery for cancer of the lung or oesophagus (PRE-HIIT): protocol for a randomized controlled trial

**DOI:** 10.1186/s12885-020-06795-4

**Published:** 2020-04-15

**Authors:** Gráinne Sheill, Emer Guinan, Linda O’Neill, Charles Normand, Suzanne L. Doyle, Sarah Moore, John Newell, Grainne McDermott, Ronan Ryan, John V. Reynolds, Juliette Hussey

**Affiliations:** 1 Discipline of Physiotherapy, School of Medicine, Trinity College, the University of Dublin, Dublin, Ireland; 2 School of Medicine, Trinity College, the University of Dublin, Dublin, Ireland; 3 Centre for Health Policy and Management, Trinity College, the University of Dublin, Dublin, Ireland; 4 School of Biological and Health Sciences, Technological University Dublin, Dublin, Ireland; 5 Physiotherapy Department, St James’s Hospital, Dublin 8, Ireland; 6 School of Mathematics, Statistics and Applied Mathematics, National University of Ireland, Galway, Ireland; 7 Department of Anaesthesia and Intensive Care, St. James’s Hospital, Dublin, Ireland; 8 Department of Surgery, Trinity Translational Medicine Institute, Trinity College, The University of Dublin and St. James’s Hospital, Dublin, Ireland; 9 Department of Surgery, St. James’s Hospital, Dublin, Ireland

**Keywords:** Exercise, Preoperative care, Prehabilitation, Fitness

## Abstract

**Background:**

Patients with cancer of the lung or oesophagus, undergoing curative treatment, usually require a thoracotomy and a complex oncological resection. These surgeries carry a risk of major morbidity and mortality, and risk assessment, preoperative optimisation, and enhanced recovery after surgery (ERAS) pathways are modern approaches to optimise outcomes. Pre-operative fitness is an established predictor of postoperative outcome, accordingly, targeting pre-operative fitness through exercise prehabilitation has logical appeal. Exercise prehabilitation is challenging to implement however due to the short opportunity for intervention between diagnosis and surgery. Therefore, individually prescribed, intensive exercise training protocols which convey clinically meaningful improvements in cardiopulmonary fitness over a short period need to be investigated. This project will examine the influence of exercise prehabilitation on physiological outcomes and postoperative recovery and, through evaluation of health economics, the impact of the programme on hospital costs**.**

**Methods:**

The PRE-HIIT Randomised Controlled Trial (RCT) will compare a 2-week high intensity interval training (HIIT) programme to standard preoperative care in a cohort of thoracic and oesophageal patients who are > 2-weeks pre-surgery. A total of 78 participants will be recruited (39 per study arm). The primary outcome is cardiorespiratory fitness. Secondary outcomes include, measures of pulmonary and physical and quality of life. Outcomes will be measured at baseline (T0), and post-intervention (T1). Post-operative morbidity will also be captured. The impact of PRE-HIIT on well-being will be examined qualitatively with focus groups/interviews post-intervention (T1). Participant’s experience of preparation for surgery on the PRE-HIIT trial will also be explored. The healthcare costs associated with the PRE-HITT programme, in particular acute hospital costs, will also be examined.

**Discussion:**

The overall aim of this RCT is to examine the effect of tailored, individually prescribed high intensity interval training aerobic exercise on pre-operative fitness and postoperative recovery for patients undergoing complex surgical resections, and the impact on use of health services.

**Trial registration:**

The study is registered with Clinical Trials.Gov (NCT03978325). Registered on 7th June 2019.

## Background

Cancer prehabilitation is an essential component of the multidisciplinary treatment model that optimises patient outcomes at each step on the continuum of cancer care, from diagnosis to survivorship [1, 2]. Prehabilitation aims to reduce post-treatment morbidity and improve pre-treatment health status to increase treatment options, typically surgical candidacy. With the evolution of multimodal approaches to cancer treatment [3], there are now multiple opportunities for prehabilitation in oncology care.

Attenuating postoperative risk is a key priority of preoperative assessment and enhanced recovery after surgery (ERAS) protocols. In lung and oesophageal cancer, complex surgical resection is the only curative intervention, however these procedures are associated with significant postoperative challenges and have the highest mortality and major morbidity risk compared to other oncologic surgeries. Postoperative pulmonary complications (PPCs), which are among the most serious postoperative morbidity, occur in 15–30% of patients post-oesophagectomy and are the primary cause of postoperative mortality, contributing to 45.5–55% of post-oesophagectomy deaths [4]. Comparably, rates of PPCs following thoracic surgery are 25% [5]. The impact of postoperative complications, particularly PPCs on long-term morbidity and quality of life (QOL) is well documented [6], and leads to significant hospital cost, driven by increased use of critical care facilities, and protracted hospital length of stay (LOS). The median cost of hospitalisation post oesophagectomy is $31,375 (€26,368), however this increases by a further $20,777 (€17,461) with serious postoperative complications [7]. Consequently, interventions aimed at reducing postoperative risk may have significant clinical and economic impact.

Cardiopulmonary fitness is an established indicator of postoperative outcome and surgical candidacy. In intra-abdominal surgery, low cardiopulmonary fitness, characterised by an anaerobic threshold (AT) < 11 ml/kg/min provides the most accurate indicator of postoperative morbidity and hospital LOS [8]. Comparably, peak oxygen consumption (VO2peak) < 10 ml/min/kg is predictive of mortality and serious morbidity following major thoracic operations [9]. While a relatively weak association is reported between cardiopulmonary fitness and oesophagectomy outcome specifically [1, 8], it follows from the robust thoracic and intra-abdominal literature that preoperative fitness is an important determinant of oesophagectomy outcome. Furthermore, as oesophageal cancer management moves towards multimodality [3], the attrition impact of preoperative chemo(radio)therapy leads to considerable physical deconditioning and loss of preoperative fitness thus compromising post-operative outcome [10]. In a multivariate analysis, a 1.0 ml/kg/min increase in fitness reduced the odds of complications following colorectal resection by over 20% (OR 0.77 (95% confidence interval (CI) 0.66–0.89) while an increase of 2.0 ml/kg/min was associated with a 40% reduction (OR 0.6 (95%CI 0.45–0.80) [11]. In intra-abdominal surgery, prehabilitation involving inspiratory muscle training (IMT), aerobic exercise and resistance training reduces the incidence of postoperative complications by 41% (OR 0.59 (95%CI 0.38–0.91), with the strongest impact observed in relation to PPCs specifically [12].

While the evidence in intra-abdominal surgery is encouraging, little is known about exercise prehabilitation for major thoracic operations on the lung or oesophagus. Preliminary results from moderate-to-vigorous intensity aerobic exercise programmes prior to thoracotomy for lung cancer have reported improvements in pre-operative cardiopulmonary fitness, and QOL, however trials are limited by single-armed designs and low participant numbers [13]. Exercise prehabilitation for oesophagectomy has typically prescribed IMT, a form of exercise training which strengthens the inspiratory muscles. While initial results from IMT trials were encouraging, a randomised trial of 246 patients, reported no reduction in pneumonia rates or PPCs following oesophagectomy with IMT [14], highlighting the need to prescribe more intensive exercise training to achieve a higher training stimulus for clinical impact.

High intensity interval training (HIIT) has significant potential as an effective and feasible preoperative intervention [15–17]. HIIT consists of alternating periods of high-intensity aerobic exercise and low-intensity exercise or rest, and stimulates superior improvements in VO2peak compared to continuous moderate-intensity aerobic training [15, 18]. The rationale for its use is to increase training time spent at a high percentage of VO2peak (> 80% VO2peak), thus producing a stronger stimulus for cardiovascular and muscular adaptations. Consequently, HIIT provides the best opportunity to create optimal improvements in fitness within the short timeframe for oncologic resection within the clinical pathway. In oncologic resection, preliminary evidence indicates 12–15 sessions of HIIT significantly improves cardiopulmonary fitness in low-fit older adults undergoing lobectomy [16] and hepatic resection [17], however further evaluation in larger cohorts and in those with highest postoperative risk is required. Pilot research from our group supports the feasibility and preliminary efficacy of the proposed intervention.

The primary aim of this study is to improve pre-operative fitness with high intensity interval training (HIIT). This study will examine the physical and economic implications of a targeted programme of exercise prehabilitation prior to major oncologic resection for oesophageal and lung cancer. The HIIT exercise programme will be considered in the context of standard clinical pathways, involving pre-operative oncological therapy and an enhanced approach to postoperative recovery, and compared to standard care.

## Methods

### Study aims

The primary aim of this work is to examine the effect of a preoperative high intensity interval training programme on cardiorespiratory fitness in patients scheduled for oesophagectomy and major lung resections.

Secondary aims are;

- To examine if individually prescribed preoperative exercise training can reduce postoperative complication rates following thoracic oncological resections.
- To determine if individually prescribed preoperative exercise training can impact post-operative physical recovery following complex thoracic oncological resections.
- To determine if individually prescribed preoperative exercise training restores preoperative cardiorespiratory fitness to pre-treatment levels in patients treated with preoperative chemo(radio)therapy and surgical resection.
- To examine if individually prescribed preoperative exercise training can reduce postoperative healthcare costs, in particular acute hospital costs.
- To qualitatively explore participant’s experience of preparation for surgery on the PRE-HIIT trial.

### Study design

**Fig. 1:**
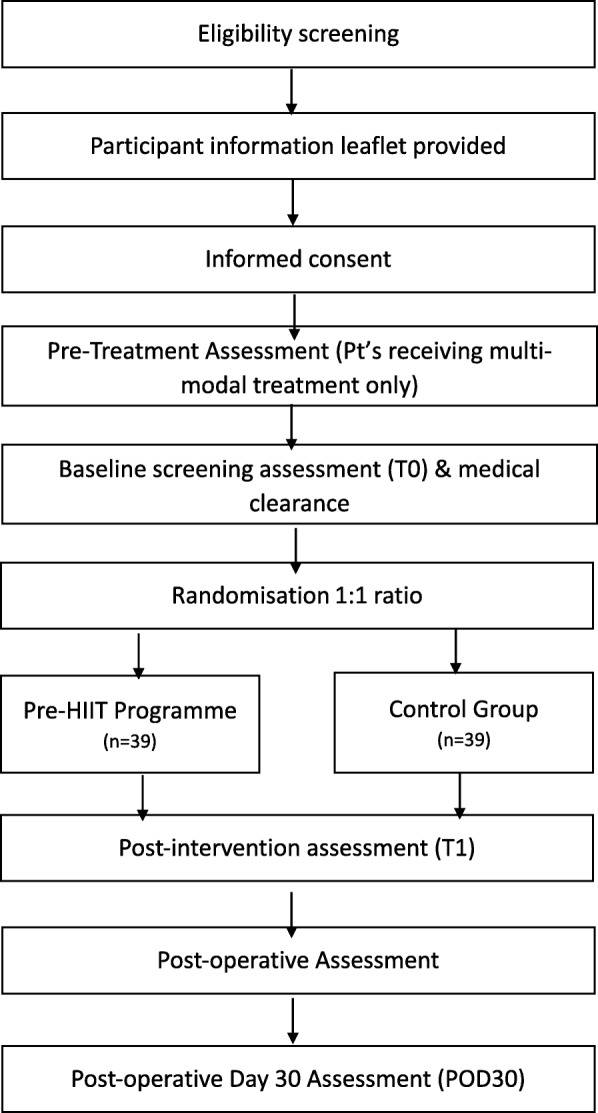
The Pre-HIIT Trial

PRE-HIIT will be implemented as a randomised controlled trial with two arms: i) an intervention group offered the 2-week HIIT programme in addition to standard care, and ii) a control group receiving standard survivorship care, which involves standard pre-operative advice and a preoperative moderate intensity exercise programme. Figure 1 depicts the flow of participants through the study. The study will take place in the Wellcome Trust-Health Research Board (HRB) Clinical Research Facility (CRF) at St James’s Hospital (SJH), Dublin. Ethical approval has been sought from the Tallaght University Hospital (TUH)/ SJH Ethics Committee. Any amendment to the protocol which may impact on the conduct of the study will be submitted as an amendment for approval to the ethics committees. The study will be performed according to the Declaration of Helsinki. The study is registered with Clinical Trials.Gov (NCT03978325).

### Study participants

Participant Selection Criteria:

#### Inclusion criteria

- Patients who are scheduled for either oesophagectomy (2-stage or 3-stage) or major lung resection for the management of primary oesophageal or lung cancer.
- Date of surgery ≥2 weeks from baseline (T0) assessment
- Ability to provide written informed consent
- Absence of significant co-morbidities, including metastatic disease, which may adversely impact postoperative outcome
- Successful completion of a medically supervised cardiopulmonary exercise test
- Patients with oesophageal cancer scheduled for multimodal therapy including preoperative chemo(radio)therapy and oesophagectomy will be recruited and tested prior to treatment commencement (Dx).

#### Exclusion criteria

The American Thoracic Society/American College of Chest Physicians (ATS/ACCP) absolute contraindications for exercise testing will be applied [19].

In addition, patients undergoing video assisted lobectomy (VATS) for early lung cancer will be excluded.

### Recruitment and screening

The PRE-HIIT trial will recruit 78 patients. Participants will be recruited from SJH, Dublin, Ireland. Participants will be identified at pre-operative clinics and through institutional databases by their clinical team. Eligibility screening will be completed by the research team at SJH. All participants will require written medical clearance from their treating consultant prior to enrolment. Potentially eligible patients will be informed about the study by a member of the research team (SJH) and will receive a participant information leaflet. Following a reflection period of 24–48 h, a researcher will telephone the patient to confirm their interest in participation. Patients who are willing to participate will be invited to attend the CRF at SJH to provide the research team with written informed consent and for baseline testing.

### Randomisation, allocation, concealment and blinding

Following baseline assessment (T0) and registration, participants will be randomised in a 1:1 ratio to either the PRE-HIIT programme or to the standard survivorship care control group, using a computer-generated randomisation list. Randomisation will be overseen by a co-principle investigator, who will have no direct involvement in implementing the trial.

Allocation order and treatment assignment will be concealed from investigators using sealed envelopes generated based on order of recruitment. Study assessments will be performed by an assessor blinded to treatment allocation. Due to the nature of the PRE-HIIT programme, neither the programme implementation staff nor patients can be blinded to the participants’ randomisation assignment.

### Intervention

#### Preoperative exercise intervention

The exercise intervention will take the form of a supervised programme, completed for at least 2 weeks, up to 5 days per week preoperatively. The feasibility of this protocol has been established by our PHIIT trial, which recruited patients scheduled for thoracotomy and colorectal surgery at St James’s Hospital, with compliance rates of 86% and adherence of 98%. Furthermore, the study design is similar to the format prescribed by the PREPARE trial [20], which specified a minimum study duration (at least 2 weeks) but continued to prehabilitate patients for longer in the lead up to surgery to optimise the benefits gained [21]. The 2-week preoperative timeline was found to be acceptable in the oesophagectomy clinical pathway at our Centre.

The HIIT programme will be performed on an electromagnetically braked cycle ergometer in the exercise physiology room at the CRF in St. James’s Hospital under the supervision of a physiotherapist with experience in exercise prescription and exercise oncology. Exercise sessions will be individually supervised and scheduled at a time of convenience for each participant. Each exercise session will last 40 min and will include warm-up, exercise training and cool-down components. Lactate threshold, measured during the baseline CPET, will be used to determine the exercise intensity.

The intervention will prescribe the same HIIT protocol used by the PHIIT trial, with established safety and feasibility. The training protocol will prescribe 15 s intervals of exercise and passive recovery. The highest resistance reached during the baseline CPET (measured in watts) will be recorded as the peak power output (PPO). During training, participants will undergo a 5-min warm-up at 50% PPO, followed by up to 30 min of HIIT with intervals of 15 s at 100% PPO with 15 s recovery periods at 0 watts. Vital signs including heart rate, blood pressure, perceived exertion, and oxygen saturation levels will be measured throughout. A 3-min cool down will be performed upon session completion followed by a passive recovery period of up to 7 min. Preliminary evidence from both our PHIIT feasibility trial and the published literature [16, 17], supports the hypothesis that this exercise prescription will produce a clinically and significantly meaningful increase in cardiopulmonary fitness.

#### Maintenance of nutritional adequacy

It is well established that cancer and its treatment can have a significant negative impact on a patient’s nutritional status. Participants randomised to the intervention will receive an additional tailored dietetic assessment with the exercise programme to ensure nutritional adequacy is maintained throughout the duration of the intervention. Sessions will focus on ensuring adequate dietary energy (25-30kcals/kg/day) and protein intake (1.25–1.5 g/kg/day). These reference levels are recommended in the ESPEN guidelines for cancer patients [22]. Assessments will be carried out by a registered dietitian with experience working with surgical oncology patients.

#### Standard preoperative care group

The standard care control group will not be invited to participate in the HIIT exercise group. They will however form an active control group, as standard preoperative care at St James’s Hospital includes standard pre-operative exercise advice and prescription of a moderate intensity exercise programme. Patients will also complete preoperative assessments as described and receive pre-operative health education.

### Measures

**Fig. 2:**
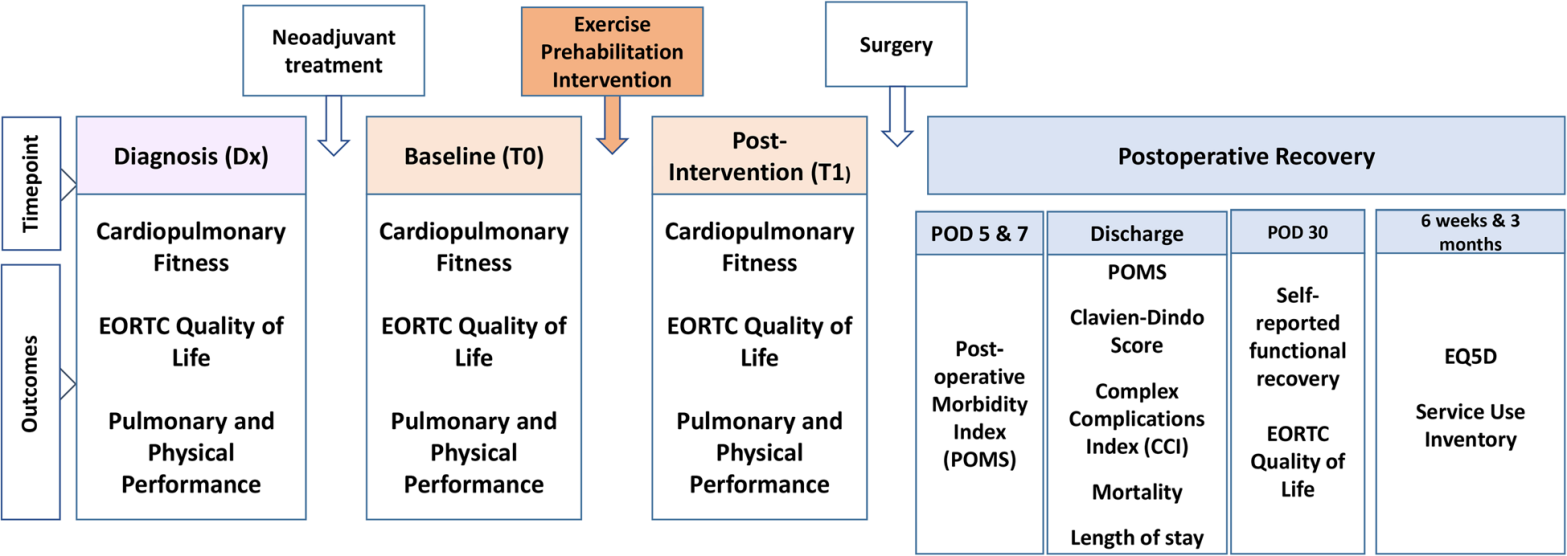
Key timepoints for assessing outcomes of exercise prehabilitation intervention

PRE-HIIT study outcomes are listed in Table 1. The main assessment battery will be performed at; diagnosis (oesophageal cancer patients scheduled for neoadjuvant treatment) (DX), baseline (T0), and post-intervention (T1). Quality of life will be further assessed at 3 months post intervention. Key timepoints for assessing outcomes of exercise prehabilitation intervention are outlined in Fig. 2. At baseline information regarding socio-demographics will be collected from patient interview and data pertaining to medical history, cancer diagnosis and treatments will be obtained from patient’s medical records.

**Table 1:** Pre-HIIT Outcomes

Outcome	Instrument	Diagnosis	Baseline	Post-intervention	Post-Operatively
		Dx	T0	T1	
**Primary outcome**					
Cardiorespiratory fitness	Cardiopulmonary Exercise Test (CPET)	X	X	X	
**Secondary outcomes**					
*Pulmonary and Physical Performance*					
Functional performance	Short Physical Performance Battery (SPPB)	X	X	X	
Muscle Strength	Leg Press 1-RM	X	X	X	
Physical activity	International Physical Activity Questionnaire	X	X	X	
Pulmonary Function	CPET	X	X	X	
Maximum Inspiratory Pressure	PowerBreathe K-series	X	X	X	
Nutritional Status	Dietary interview	X	X	X	
Quality of Life	EORTC-QLQ-C30	X	X	X	X
Cancer specific quality of Life	EORTC-QLQ-OG25 (oesophago-gastric cancer)	X	X	X	X
	EORTC-QLQ-LC 13 (lung cancer)	X	X	X	X
Qualitative approach	Semi –structured interviews (focus groups or 1:1)			X	
Cost analyses	EQ5D				X
	Service Use Inventory				X
**Post-operative morbidity**					
Post-operative outcomes	Self-reported Functional Recovery				X
	Post-Operative Morbidity Score				X
	Clavien Dindo Score				X
	Comprehensive Complications Index				X
**Other**					
Adherence	Record in case report form/ exercise diary			X	
Sociodemographic details	Participant self-report		X		
Body composition	Anthropometry		X	X	
Cancer/Surgery history	Medical records		X		
Adverse events	Reports of patients/ research personnel			X

### Primary outcome

#### Cardiopulmonary fitness

Cardiopulmonary fitness will be determined, by a maximal cardiopulmonary exercise test (CPET), the gold standard measure of fitness [23]. Cardiopulmonary exercise tests will be performed in the CRF at St James’s Hospital under medical supervision at Dx (oesophageal cancers only), T0 and T1. The CRF at St James’s is equipped with an electromagnetically braked cycle ergometer (COSMED, **E200 P/K)**, COSMED K4b2 ambulatory metabolic system, integrated exercise ECG and integrated blood pressure monitoring. The CPET will be performed using a ramp cycle ergometer protocol. Following a standardised resting and warm-up period, testing will commence with 3 minutes of free-wheel pedalling after which resistance will increase by standardised increments. The ramp gradient will be set to 10–25 W/min based on the equation below by Wasserman et al. [24].

i. VO_2_unloaded in ml/min = 150 + (6 x weight kg)
ii. Peak VO_2_in ml/min = (height cm – age years) × 20 (sedentary men) × 14 for sedentary women
iii. Work rate increment minute/watts = (peak VO_2_ml/min – VO_2_unloaded ml/min)/100)

Breath-by-breath gas analysis, heart rate, heart rhythm (12-lead ECG), non-invasive blood pressure, oxygen saturation, rate of perceived exertion and blood lactate will be measured before, during and after testing. The test will be terminated by a cool-down performed at a resistance of 30 watts for 3 minutes and during which participants will be monitored for signs of distress (pallor, chest pain, dyspnoea). VO_2_peak will be calculated as an average over the last 30 seconds of exercise. AT will be described as lactate threshold (LT) and ventilatory threshold (VT). Lactate Threshold (LT) will be measured via ‘a pin-prick’ blood sample, analysed using the ‘Stat Strip Xpress LAC’ portable lactate monitor. Samples will be collected and recorded every minute during the exercise test. Post completion of the test, the results will be inputted on a graph against time/work rate. The LT is the point in which VO_2_ increases non-linearly, this will be calculated via visual inspection, a valid method of determining LT. VT will be determined through the modified v-slope method, which determines the point of the change in slope of the relationship of VO_2_above which VCO_2_ increases faster than VO_2_ without hyperinflation [19]. Other components of fitness that will be obtained from breath-by-breath analysis include: VE/VCO_2_, VE/ VO_2_, minute ventilation.

### Secondary outcomes

#### Secondary outcome 1: postoperative morbidity

Postoperative morbidity will be measured using a suite of validated instruments; the Clavien-Dindo Scale, the postoperative morbidity index (POMS) and the Comprehensive Complications Index (CCI)**.** These widely used objective measures of postoperative complications, overcome the known issues with standardising the definition of postoperative complications [4]. In addition, hospital and critical care LOS and postoperative mortality will be recorded. All outcomes will be recorded on hospital discharge and on POD30 with the exception of the POMS which will additionally be recorded on POD3, POD5 and POD7 (Table 1).

##### Clavien-Dindo scale

The Clavien-Dindo classification score measures the most severe complication that occurs in the postoperative period. The ordinal scale is divided into seven grades (Grade I-V, two sub-groups each for Grade III and IV), ranging from Grade I, which considers any deviation from normal, to Grade V, which describes death of the patient. The tool is widely used due to its simplicity and reproducibility, its correlation with LOS, and the degrees of severity of complications also correlate with the perceptions of severity of medical staff and patients [25].

##### The post-operative morbidity score (POMS)

The POMS is a nine-domain tool that prospectively describes and records in-hospital postoperative complications following major surgery [26]. The POMS, which is performed on specific postoperative days, typically POD3, POD5 and/or POD7, provides defined criteria for complications in nine categories classified by organ system including pulmonary, infectious, renal, gastrointestinal, cardiovascular, neurological, haematological, wound and pain.

##### The comprehensive classification index (CCI)

The CCI provides a summary of overall morbidity including the type, number and severity of each complication experienced during the postoperative period [25]. When compared to the Clavien-Dindo Classification the CCI demonstrates a superior ability to discriminate between patients with a different number and severity of complications.

### Secondary outcome 2: pulmonary and physical performance

The training stimulus associated with HIIT may lead to important improvements in secondary measures of physical performance including pulmonary function, ventilatory and peripheral muscle strength and functional capacity [27], which may in turn positively impact postoperative outcome. The following measures will be performed at Dx, T0 and T1. Self-reported functional recovery will be evaluated on POD30.

#### Pulmonary function

Pulmonary function will be measured as the first step of the CPET and analysed as a secondary outcome. The COSMED K4b2 ambulatory metabolic system will be used to determine forced vital capacity (FVC), forced expiratory volume at 1 s (FEV1) and the ratio of FEV1/FVC in accordance with the American Thoracic Society Guidelines.

#### Maximal inspiratory pressure

Maximal inspiratory pressure (PImax) provides a non-invasive, simple measure of inspiratory muscle strength, particularly the diaphragm. PImax will measured using a PowerBreathe K-series portable respiratory pressure metre. Patients will be measured at residual volume during a forceful inspiratory manoeuvre while resting in a seated position. All measures will be performed in triplicate with the best measure taken for data entry.

#### Peripheral muscle strength

Preliminary analysis of the PHIIT trial participants has demonstrated a post-intervention increase in peak power output, suggesting some improvement in lower limb strength with the cycle ergometer HIIT protocol. In the proposed study, quadriceps muscle strength will be measured by 1 repetition maximum (1RM) using a horizontal leg extension. The 1RM is defined as the highest load that can be lifted through full range of movement at one time. Participants will complete 1RM testing following adequate aerobic warm up followed by a warm up of 6 repetitions at 60% 1RM with 2 min rest and then 3 repetitions at 80% 1RM with 2 min rest. A maximum of 5 trials to determine 1RM will be completed with a rest period of 2 minutes between each trial.

#### Functional performance

This will be measured using the Short Physical Performance Battery (SPPB). This measure combines the results of gait speed, chair stand and balance tests. A score lower than 10 indicates one or more mobility limitations.

#### Self-reported physical activity

Physical activity will be measured subjectively using the self-administered International Physical Activity Questionnaire (IPAQ). The IPAQ long form comprises four activity domains (work, leisure, transportation and household) which evaluates activity in metabolic equivalent (MET)-hours per week over the previous 7 days. The questionnaire also quantifies average weekend and weekday sitting time. Data collected on POD30 will be collected via a telephone call with the participant.

#### Self-reported functional recovery

Patient perceived physical recovery at POD30 will be self-assessed using standardised classifications. Participants will rate their recovery as 0, 25, 50, 75% or 100% according to standardised descriptors [28]. Data collected on POD30 will be collected via a telephone call with the participant.

### Secondary outcome 3: quality of life

The EORTC QOL questionnaire is an integrated system for assessing the health related QOL of cancer patients participating in international clinical trials. The core questionnaire, the QLQ-C30 has been used in a wide range of cancer clinical trials [29]. It is supplemented by disease specific modules. Categories include functional scales, global health status and QOL scale, in addition to several single-item symptom measures. Disease specific modules such as the oesophageal EORTC QLQ-OES18 and QLQ-OES25 and the lung EORTC QLQ-LC 13 can be used in conjunction with the main questionnaire to monitor disease specific symptoms. In the current study, QOL will be measured using EORTC QLQ-C30 and relevant subsets at Dx, T0, T1 and on POD30.

QOL will be further measured using the EuroQol (EQ) EQ-5D5L to analyse cost effectiveness of the intervention. The EQ-5D5L comprises five dimensions: mobility, self-care, usual activities, pain/discomfort and anxiety/depression with each dimension rating activities as having no problems, having slight problems, having moderate problems, having severe problems and being unable to do/having extreme problems. The EQ-5D5L will be measured at routine post-operative clinic visits at 6 weeks and 3 months postoperatively.

Changes in the QOL scores (both from EORTC QLQ and EQ5D5L) will be analysed to identify any difference in the profile of health related QOL in the two arms of the study. If costs of hospital care are lower in the intervention group, and if QOL scores are better post-intervention, then the intervention will dominate (ie, lower costs of hospital care, including the cost of the exercise programme, and better QOL of patients) and no cost-effectiveness ratios can be calculated. Otherwise it will be possible to estimate the additional cost of any measured improvement in QOL post-intervention. Similarly, if costs of care are lower in the intervention group but QOL is also lower, it will be possible to estimate cost-effectiveness ratios for this change.

### Qualitative approach

At the pre-surgery assessment (T1) a sub-cohort of the study’s participants will take part in a semi-structured interview to feedback on how the PRE-HIIT study has impacted on their preparation for surgery. Interviews will be held with approximately 20 participants or until data saturation is reached.

### Adherence

Adherence to the exercise component of PRE-HIIT will be measured with traditional adherence variables i.e. attendance at supervised sessions and completion of home-based sessions and monitoring of compliance to the prescribed exercise protocol. Compliance to the aerobic component will be documented by the achieved heart rates on the Polar Heart Rate Monitors, and the duration of aerobic exercise, and for resistance training, the weight, number of sets, and repetitions will be recorded. During the supervised sessions compliance will be monitored by the supervising physiotherapist, whilst during homebased sessions participants will record their compliance in a home exercise diary. PRE-HIIT will implement a series of drug trial adapted adherence outcomes as described by Nilsen et al. [30]. These additional exercise adherence variables will include; permanent treatment discontinuation, treatment interruption, dose modification, early session termination, and pre-treatment intensity modification. Adherence variables are described fully in Table 1.

### Hospital resources

The costing of hospital stays, and interventions will be carried out based on activity data from hospital records, with unit costs taken from the standard estimated costs from the Healthcare Pricing Office. Programme implementation costs will be analysed in consideration of clinician salaries, overheads and equipment costs. Formal care costs will be extracted from medical charts and from the institutional database in consideration of preoperative characteristics, surgery type and postoperative recovery including complications. Since the participants in the study are randomised to each arm, the comparison of costs of hospital stays will be reported as differences and the normal tests of significance. It is also important to assess if the intervention has longer term effects beyond hospital discharge. The destination at discharge, use of community health services from the time of discharge to the follow up outpatient appointment and EQ5D5L scores at the time of the follow up appointment will be collected for each participant. This will allow the measurement of any effects of the intervention on the feasibility of the patient going home directly from hospital, and any difference in the need for community health services. The EQ5D5L scores at outpatient follow up will allow an assessment if any short-term difference persists and will assist in modelling the likely medium-term effect of the intervention on costs and QOL.

### Safety

Prior to baseline testing, all participants will require written medical approval confirming their suitability for participation. Patients will only be formally enrolled on the study after successfully completing a CPET with ECG monitoring. All CPET will be medically supervised and will take place in the CRF which is located within SJH and is covered by the hospital’s emergency response team. All adverse events will be recorded, and serious adverse events will be reported to the research ethics committees.

### Sample size calculation

The primary response is the change in VO_2_peak from baseline (T0) to post-intervention (T1). On the basis of estimates calculated from our pilot study, a sample of size 64 (32 in each arm) is needed in order to detect a mean difference in VO_2_peak of 1 ml/kg/min between the control and intervention groups (assuming a standard deviation of change in VO_2_peak of 1.4 ml/kg/min for each arm) with 80% power at the 5% significance level based on a two-sample t-test. Based on the results from the PHIIT trial and others [16, 17], an improvement of 1 ml/kg/min in VO_2_peak is feasible with the exercise intervention prescribed. It is anticipated that the active control group will not experience a change in fitness during this 2 week time period [16]. Moderate intensity exercise interventions have not been shown to significantly change preoperative aerobic capacity and functional capacity of patients due to undergo elective surgery [31].

### Statistical analysis

Quantitative data analysis will be performed using R IBM SPSS software, employing statistical best practice. A comparison of patient characteristics at baseline will be carried out for each arm. Summary statistics for continuous variables (means and standard deviations or median and ranges as appropriate) and categorical variables (counts and proportions) will be presented. Graphical summaries (boxplots, case profile plots, labelled scattered plots) will be used to compare the distribution s of each response variable and for patient characteristics between the arms. A linear mixed model will be used to model the longitudinal change in the primary response between the groups, allowing for missing data (under the assumption that data are missing at random) and allowing for within subject correlations in the repeated measures across time. The model will adjust for the baseline response variable and other covariates as necessary.

A qualitative approach will be taken to gather participants’ feedback on how the PRE-HIIT study has impacted on their preparation for surgery. Data collection will take a semi-structured approach individual interviews following the intervention. The discussion guide for the interviews will explore topics such as: the impact of the intervention on health, well-being, and activities of daily living, facilitators and barriers to preoperative exercise, and recommendations for future implementation of the programme. Interviews will be digitally audio-recorded and transcribed verbatim for data analysis. A qualitative descriptive approach [32] will be taken to the analysis, with the aim of providing a substantial description of what the participants said, without drawing deep implications from the data. Braun and Clarke’s 6 stage approach to thematic analysis will be used to analyse all data collected [33]. A team of researchers will analyse all transcripts following an agreed process using nVivo 12 (QSR International, Australia).

### Data monitoring

Data monitoring will be provided by the trial steering committee, including overall project supervision, progress monitoring, advice on scientific credibility and ensuring the integrity and appropriate running of the project. The research team will make quarterly reports to the trial steering committee.

### Dissemination

Findings of PRE-HIIT will be disseminated via peer-reviewed publications and conference presentations. Aggregate study results will be presented to participants and their families at an education symposium upon study completion. Anonymised data will be made available on an open access repository.

### Public and patient involvement (PPI)

PRE-HIIT will involve a number of PPI initiatives. We will seek feedback on participant documentation, particularly the participant information leaflet and consent form, to ensure readability and clarity. In addition, a patient representative will be invited to speak at the education symposium in the final year of the project.

### Study status

PRE-HIIT will begin in recruitment in 2020.

## Discussion

The PRE-HIIT RCT will examine the influence of pre-operative high intensity interval training programme on physiological outcomes and postoperative recovery and, through evaluation of health economics, the impact of the programme on hospital costs**.**

There is a need for clinically feasible interventions that attenuate the impact of multiple therapeutic interventions, most particularly major oncological operations, and accelerate patient recovery. Prehabilitation is likely to have its greatest impact in cancer populations who experience the greatest treatment morbidity, such as oesophageal and lung cancer. There is growing interest in the value of exercise prehabilitation to increase preoperative fitness above critical values in oncologic resections [15]. HIIT exercise training stimulates great improvements in cardiopulmonary fitness over short periods compared to continuous aerobic training and therefore may be ideally suited to exercise prehabilitation. This mode of exercise prehabilitation may attenuate postoperative risk and improve postoperative recovery, thus improving patient quality of life and having considerable economic benefits for the healthcare system. Given the high unit cost of hospital days and procedures, the direct effects of the intervention on hospital costs and any evidence of differences in QOL which will be explored by the PRE-HIIT trial are of considerable interest. The results of this study will inform current perioperative practice and will provide direction for future research.

## Data Availability

Not applicable.

## Abbreviations

CPET: Cardiopulmonary exercise test
CRF: Clinical research facility
ERAS: Enhanced recovery after surgery
HIIT: High intensity interval training
LOS: Length of stay
LT: Lactate threshold
POD30: Post-operative day 30
PPI: Public and patient involvement
QOL: Quality of life
RCT: Randomised controlled trial
VATS: Video assisted lobectomy
VT: Ventilatory threshold
